# The Proofs of Infanticide Considered

**Published:** 1837-07

**Authors:** 


### ART. VI.

1. *The Proofs of Infanticide, considered.* By WILLIAM CUMMIN, M.D.; Member of the Royal College of Physicians, &c.; and Lecturer on Forensic Medicine at the Aldersgate School of Medicine.—*London*, 1836. 12mo. pp. 95.

2. *Beiträge zur Lehre von dem Thatbestande des Kindermordes überhaupt, und den ungewissen Todesarten neugeborner Kinder insbesondere.* Von IGNAZ. SCHWÖRER, Doctor der Medicin, ordentl. öffentl. Professor der Geburtskunde an der Universität Freiburg, &c. —*Freiburg*, 1836.

*Contributions to the Doctrine of Infanticide, more especially in Relation to the doubtful Causes of Death in New-Born Children.* By IGNATIUS SCHWÖRER, M.D., Professor of Midwifery in the University of Freiburg, &c.—*Freiburg*, 1836. 8vo. pp. 45.

3. *Researches in Medicine and Medical Jurisprudence.* By JOHN B. BECK, M.D., Professor of Materia Medica and Medical Jurisprudence in the College of Physicians and Surgeons of New York, &c.—*New York*, 1835. 8vo. pp. 256.

THERE are perhaps few among the present race of practitioners who are not acquainted with the opinions of Dr. William Hunter on the subject of infanticide. The influence of his name sufficed, for a very considerable period, to retard the progress of enquiry relative to one of the most important and embarrassing of the medico-legal questions which present themselves for investigation in cases of child-murder. The tract by which the opinions of Dr. Hunter were made known to the profession, was not published until some time after his death; and we are inclined to agree with Dr. Cummin, who has very judiciously reprinted it, with comments and observations, that, had the author lived to superintend its publication, it would have undergone some material alterations before being submitted to the public. As it is, "Where shall we find," observes Dr. Cummin (p. 27), "a more splendid defence of the sex, or one more humanely indulgent to those unfortunate females who happen to lie under the imputation of the murder of their offspring?" No one can dispute the eloquence or the ingenuity of Dr. Hunter; but it is always to be regretted when these excellent qualities in a scientific writer are made to serve a bad purpose, and one which he never could have contemplated,—namely, that of inventing a specious defence for the really guilty. That this has been the case, and that the strongest prejudices have been exerted against a very useful, and, when properly conducted, a very safe method of determining the all important question, whether the child which is the subject of enquiry has lived to breathe, will be apparent, on reference to any volume of our Criminal Law Reports. "The judges," remarks Dr. Cummin, in his preface, "quote it (Dr. Hunter's tract,) with implicit faith in its perfection: the bar study it, and cross-examine the crown-witnesses on the difficulties which it suggests; and medical men, probably, will not find it safe to venture into the witness-box without being familiarly acquainted with its contents." This is no exaggerated picture: we could at this moment refer to several excellent works on our criminal law, which have but recently appeared, in which Dr. Hunter's opinions are exclusively adopted, and the whole subject is disposed of as if nothing had been done since the time of that distinguished writer. How necessary is it, then, that a medical practitioner should be prepared with a knowledge of doctrines and opinions to which so much importance is attached by our law-authorities!

Dr. Cummin has divided his treatise into three parts. In the first, we have a reprint of Dr. Hunter's tract "On the Uncertainty of the Signs of Murder in the case of Bastard-children:" in the second, we have comments upon this tract; and in the third, we have a compendious summary of our author's own views on the subject of infanticide, including the most important facts which have of late years been accumulating in this interesting department of Forensic Medicine.

In his comments upon Dr. Hunter's opinions, Dr. Cummin has very properly confined himself to a notice of those which are the most objectionable and are most likely to mislead non-professional readers. In giving to Dr. Hunter credit for humanity and ingenuity, he does not hesitate to expose boldly the rhetorical artifices, inconclusive reasonings, and unapt illustrations with which the tract abounds. Thus, (p. 28,) Dr. Hunter complains of too much being in general left to the decision of medical practitioners; but, "instead of giving any instance of mistakes committed by medical men," he immediately quotes an example of gross ignorance on the part of a *jury*, who were only prevented from falling into a serious error by the confidence which they put in a cautiously expressed *medical* opinion! The vagueness of Dr. Hunter's language is often so great, that it is difficult to arrive at his meaning. Thus, he speaks of judging "of the death of a child, by attending to the force of cohesion between the skin and scarf-skin," (p. 30,) leaving it uncertain whether evidence as to the *period* or as to the *manner* of death is to be thereby obtained. We must refer our readers to Dr. Cummin's judicious strictures on this passage.

Undoubtedly, the most important part of Hunter's tract, and one which required the closest examination by a commentator, is that which refers to the objections brought by him against the employment of the hydrostatic test, as a means for determining whether a child has lived to breathe. Dr. Cummin has here selected for remark a question proposed by Hunter, which gives him an opportunity of entering fully into the exceptions taken by this writer to the hydrostatic test: but, as our author properly observes, "these exceptions are little more than hinted at or merely stated, inasmuch as there is nothing suggested by which their force may be duly estimated." Dr. Hunter's question is, "How far may we conclude that the child was born alive and *probably murdered by its mother*, if the *lungs swim in water*?" It would seem from the form in which this question is put, that, in Dr. Hunter's time, the swimming of the lungs in water was received as evidence of the *murder* of the child: but the facts are most unfairly stated. We are well aware that the buoyancy of the lungs was then regarded by many as an infallible sign of a child having lived; and that but little attention was paid to the other causes besides respiration, which are well known to render the fœtal lungs buoyant: but we do not think it would be easy to produce a single case in which "the swimming of the lungs" was made *evidence of murder* against the mother. All that the practitioner attempted to determine by its application was, whether the child had lived to breathe, leaving the question of murder to be settled by other evidence. It is very true that, had the statute of James I., regarding child-murder,* been rigorously enforced, many females must have been found guilty of the crime, even when the medical evidence proved no more than that the child had breathed; yet it is well known that every fiction which the law could devise to do away with the alleged concealment of pregnancy and delivery, was admitted in favour of the prisoners. Besides, even had it been otherwise, the most proper course to have pursued would have been to show the inhumanity and cruelty of a statute which called for proofs of innocence not only unnecessary, but such as the accused party could seldom be in a condition to afford. The mischievous effects of the law, however, were not noticed: an attempt was made to remedy the evils to which it gave rise, by rendering medical evidence on such investigations so uncertain, through specious objections, as to lead in general to its total rejection in a court of justice.

One of the exceptions taken by Dr. Hunter relates to the generation of air by putrefaction within the pulmonary tissue; he also proposes a method for determining whether the buoyancy be due to this cause or not. His method is "simple, but not as practical as it might be;" and our author then enters into a very concise, and at the same time a very clear, account of the means by which a medical witness may best satisfy himself of the nature of the cause to which the buoyancy is due. The same remark applies to the comments on the objection founded on the possibility of the lungs having been artificially inflated with air in the attempt to resuscitate a child.

We shall make no observation on the various "facts" adduced by Dr. Hunter, in which he attempts to account for the death of the child from natural causes. His inferences are wholly inapplicable to the present state of our law. The tendency is indeed now rather the other way; for there is not an instance in which a woman is convicted of the crime of child-murder, unless her guilt be obviously and clearly made out. The accidental and natural causes of death are now well known; and females often escape, although there may be the strongest presumptive evidence against them.

We think we cannot do better than sum up these remarks on Dr. Hunter's tract in our author's own words; and we trust that they will serve as a solemn warning to those who still prefer sheltering them-

* Stat. 21. Jac. 1. c. xxvii. By this statute, a woman, concealing the birth of her *bastard* child, was considered as having murdered it, unless she could prove that it was born dead. This statute was repealed about twenty years after Dr. Hunter's death, by the 43 Geo. III. c. lviii.

selves under the authority of his, in other respects, deservedly great name, to the rigorous investigation of facts and opinions.

"It is no doubt the duty of all to save, when in their power, the innocent from unmerited ignominy and punishment. But there is also another duty which Dr. Hunter on the present occasion seems to have wholly lost sight of; namely, that of bringing the guilty to deserved condemnation, by stripping them of the assumed garb of innocence, which would otherwise be instrumental in their escaping justice. But what, after all, has Dr. Hunter done? His avowed object was to impress the public mind to rescue, if possible, some unhappy and innocent women. He has accordingly shown in his paper, that a woman *may* be innocent, although the presumption of her guilt amount to a high degree of probability. He has ingeniously proved that most of the circumstances connected with the *floating* of the lungs may be accounted for without supposing the child to have been murderously treated. He has dexterously stated, in his own peculiar way, and with all the weight attaching to his high reputation, every objection he was acquainted with *against* the hydrostatic test. Such is the amount of public duty which our author has in the present instance discharged." (P. 44.)

Again, a little further on, he observes:

"But it is evident that his (Dr. Hunter's) resolution was deliberately formed: his purpose was to show, as strikingly as possible, the *un*certainty of the signs of child-murder; and this he has done with all the skill and ingenuity of a special pleader. It is only to be regretted that it is so often forgotten by those who adopt his opinions how very special and one-sided is his mode of treating the subject; and, above all, that the great name and authority which he still enjoys should have so long and so effectually screened the *demurrers* in his pleading."

In the concluding part of his treatise, Dr. Cummin has collected and arranged the facts relative to infanticide in a systematic form; and he has also supplied those deficiencies which necessarily occurred in the former part of his work, while he was confining himself to the particular line of argument followed by Dr. Hunter. The first question examined by our author relates to the establishment of the fact of the child having been born alive. The signs of maturity; the signs of a child having survived its birth, under which head we have a concise account of the successive changes that take place in the umbilical cord after birth; and, lastly, the indubitable signs of still-birth, with a description of the changes induced by uterine putrefaction, are treated in an able and practical manner.

The next question which Dr. Cummin proposes for examination is, *Has the infant respired?* The proof of respiration undoubtedly furnishes the best evidence of *life*, but not of a child having been *born alive;* for these, although frequently confounded, are two distinct questions. We attach but little importance to evidence founded on the external form of the chest. This cavity is commonly described as ample and arched in the child which has respired; while the contrary conditions are stated to be met with in children that have not performed the act of respiration. But it is not often that any difference of form is observable, when the child has perished soon after birth, notwithstanding that it may even have lived to respire *several hours;* and, therefore, evidence of this kind is not to be obtained in the majority of cases of infanticide. We should doubt whether the change in the form of the thorax would be at all appreciable in any instance in which the child had died within twenty-four hours after birth: and the reason for this must at once suggest itself in the fact that respiration is only slowly established; and that many hours must in general elapse before the lungs can acquire their full expansion.

We pass over the evidence derivable from the colour, volume, consistence, and absolute weight of the lungs, to that which is furnished by the hydrostatic test. In the directions which are given by many experimentalists for the employment of this test, we find it frequently recommended to remove the lungs with the heart and thymus gland attached, and to place these organs together in the vessel of water. We cannot exactly see the necessity for operating with the heart and thymus in this experiment, since the buoyancy of the lungs must be afterwards separately determined; and the degree of buoyancy which the organs possess may be much more accurately ascertained, if necessary, by attaching to them a weight. The heart and thymus vary in weight, from different causes, in different children; nor does there appear to be any close connexion between their weight and that of the lungs. If the leaving of these organs attached be superfluous under complete respiration, it becomes highly objectionable when the child has but feebly respired, or when the lungs are in a putrefied state. So, again, it is recommended that the pulmonary vessels should be secured by ligatures before removing the lungs from the chest; but this, we contend, is not required, except when we wish to ascertain with accuracy the *absolute weight* of the organs. The confining of the blood within the lungs by the application of ligatures to the pulmonary vessel, must, in cases where the act of respiration has been but feebly performed, rather interfere with the results which the experimentalist has to look for. In no case, indeed, in which the hydrostatic test is employed, do we see the necessity for resorting to this proceeding.

Several objections, it is well known, have been raised against the inferences derivable from the performance of this experiment. Among these, we shall first refer to what has been termed an *emphysematous condition* of the lungs, not depending on putrefaction or on the reception of air by respiration. According to the French medical jurists, this state occurs when a child is delivered by the feet; and when, owing to narrowness of the outlet, the chest becomes forcibly compressed. The child, in such a case, may die before delivery is complete, without respiring; and its body may be examined before putrefaction has begun; yet, says Lecieux, from whom this statement is taken, the lungs will float in water. The cause of the production of air vesicles in the pulmonary tissue, under these circumstances, is thus explained: by the compression of the thorax, the lungs are contused, blood is extravasated, which undergoes an *alteration*, by which air is afterwards extricated. This condition of the fœtal lungs is alleged to proceed from the same cause, as the emphysematous tumefaction, which sometimes follows a wound or contusion of the head. We have no hesitation in rejecting this explanation altogether: it is possible that air vesicles may be occasionally met with in the lungs of children when delivery has been accomplished in the manner stated; but, supposing respiration not to have been imperceptibly performed, the occurrence of which is not unlikely, we should ascribe their origin to incipient putrefaction. This process, it is well known, always takes place rapidly in parts which have suffered from contusion or violence. Indeed, the admission that the blood undergoes *an alteration* after it has become extravasated sufficiently bears out this view of the case. But, whatever explanation we may adopt, we must remember that the mechanical compression of any of the soft organs of the fœtus cannot, without ulterior putrefactive changes, give rise to an extrication of air within the parenchyma. This emphysematous condition of the lungs is then, in our view, nothing but ordinary putrefaction, supervening perhaps more rapidly in this than in other cases. We have thought it right to make these remarks, since there are enough real difficulties for a medical jurist to encounter in infanticidal investigations, without allowing others unnecessarily to embarrass him.

The distension of the fœtal lungs by the artificial introduction of air has been generally mentioned as a great obstacle to any correct inference from the employment of the hydrostatic test, since the time of Dr. Hunter. This is certainly one of the most specious objections which can be offered; but there are means, easy of application, by which the difficulties arising from it may be removed. These are well stated in Dr. Cummin's treatise; and we fully agree with him in opinion when he urges that, on the occurrence of this objection, the moral evidence in the case should be closely sifted. Thus he observes:

"Such a plea on behalf of an accused mother is extremely rare, not only because in most cases it has no foundation in truth, but because, to render it probable, evidence of many collateral facts must be forthcoming. The female who would endeavour to save her child by inflating its lungs, should have given other proofs besides of her maternal tenderness: she should not have concealed, at least from some intimate friend, the fact of her pregnancy; her delivery should not have been secret (?); she should have prepared for the birth,—the living birth—of her infant; there should be no marks of wilful violence on the body: in short, it is easy to judge from the history of any given case whether the accused *wished* the life or death of the child; and, therefore, whether it is likely she *would* (even allowing that, with sufficient strength and self-possession at such a moment, she *could*) inflate the infant's lungs." (P. 63.)

We do not coincide in opinion with our author, in considering that the fact of a child respiring during delivery is an objection to the hydrostatic test. We are well aware that this is commonly maintained; but let us consider the point. All that a medical jurist can truly derive from an application of this test is the knowledge whether respiration has or has not been performed; not when the process may have begun, nor whether the child respired *before* its entire birth or *afterwards*. A little reflection will show, that it is impossible to decide, from the mere buoyancy of the lungs in water, when respiration took place, except perhaps in those instances in which the act has been most fully and completely accomplished. Here, it is true, a *presumption* may arise that the child has breathed after birth; but these cases are the exceptions to the general rule. How, therefore, can that condition be urged as an objection to a test which is entirely beyond the limits of its application? It would be as reasonable to object to the use of reagents in the detection of a poison in the stomach, because we cannot learn from their employment whether the poison was received into the organ before or after death!

How, then, is a medical jurist to determine *when* the act of respiration was performed, whether *before* or *after* entire birth? The truth is, there is no test which will here assist him. We have already hinted above that the degree to which respiration has taken place may sometimes establish a presumption; for it is not usual to find the lungs possessing the same intensity of colour, weight, crepitation, and buoyancy, when the child has respired and died during delivery, as when it has survived its birth. Some have imagined that the relative size of the body of the child and of the pelvis of the mother would furnish evidence as to whether respiration had been performed before birth or not: if, it is contended, the body of the child were small, and the female outlet large, it is probable that delivery would be suddenly and rapidly accomplished. Hence, should the hydrostatic test indicate that the child had respired, the examiner might, in such a case, infer that respiration had certainly taken place after birth. But, even here, the woman being usually delivered in secret, a medical opinion could only be presumptive; and, in cross-examination, the witness would be compelled to admit that, in the best-formed pelvis, and under the most favorable delivery, there might be a sudden cessation of the pains after protrusion of the head, which would give sufficient time for the performance of respiration by the child before the entire birth of its body. It is very true that, in such a case, the death of the child before delivery from *natural* causes is very improbable; but that is not the question; the witness has first to establish the fact of the child having survived its birth. So, again, an extreme degree of buoyancy in the lungs is not an infallible criterion of respiration after birth. We have seen the lungs of a child, that had respired and died in the act of birth, float with the heart and thymus gland attached, and present the usual physical appearances of lungs which had respired after birth. Now, a reliance upon the physical characters of the lungs, without a knowledge of the facts, would probably in this case have led a medical jurist to declare that the child had breathed after it was born. To take a converse instance: it will be easily understood that a child may perform the act of respiration so feebly after its birth, as that the lungs will be scarcely altered in physical character, except in relation to their buoyancy. This is a condition of which we have seen frequent examples. The lungs were exactly in the state in which they are found in children that have breathed in the passage and died before entire delivery. These instances, then, will show that a positive opinion can rarely, if ever, be expressed as to whether a child breathed while its head merely protruded from the outlet, or after its body was entirely brought into the world.

We have been led to make these remarks because we think there is no question of greater perplexity which a barrister can put to a medical witness on a trial for infanticide, than that which involves the exact period at which the child breathed, supposing the fact of respiration to have been established by the clearest evidence. This question is now the more likely to be put, since it has been held by several of our judges that *the proof of the mere act of respiration, unless it be established that that act was performed after entire birth,* is not sufficient evidence of a child having been born alive. Dr. Cummin alludes to this decision; and quotes the case of K. V. Simpson, at the same time remarking that "a question of this kind, if admitted in every instance, would put a stop to all judicial investigations respecting infanticide." (P. 40.) In another case, that of R. V. Poulton, it was also laid down as necessary to be proved that the child was *alive after the whole of its body was in the world.* By a recent decision of Judge Parke, in the case of R. *v.* Enoch, it was held that there must be *an independent circulation* in the child before it can be accounted alive; but it is not stated what is meant by an independent circulation. Can this be considered as established before the division of the umbilical cord? If the question be answered in the affirmative, then it follows that, in the opinion of one judge, *entire birth* is not necessarily required to be proved; because the circulation in the child becomes independent of that of the mother the moment that respiration begins; and we well know that this process may commence and continue for some time before delivery is complete. Let us, however, suppose that an independent circulation, in its legal signification, is not considered to exist in the child until after its birth and *entire separation* from the mother. In this case, a medical witness will have to encounter a still greater difficulty in giving his evidence; for how is he to ascertain whether any fatal injuries, met with on the body of a child, were inflicted before or after the separation or laceration of the umbilical cord? Yet, according to this interpretation of the law, he would be required to speak to their probable infliction after the division of the cord; for, prior to this absolute separation of the child from the mother, it might be held that there was not an independent circulation in the child; and, therefore, that it had not, in law, been born alive! We shall only observe, if proof of this kind were insisted on, we do not see how any charge of infanticide could ever be sustained, except where the child was actually destroyed before eye-witnesses. Upon this singular decision, in the case of R. *v.* Enoch, it will be seen, either that it must be altogether unnecessary to prove that a child was completely born; or, on the other hand, the proof of entire birth can be of no avail, unless, conjointly with this, the witness is prepared to show that the child was wholly separated from its mother when it received the violence which caused its death. How can such conflicting opinions be reconciled with any sound administration of the criminal law!

In discussing these legal technicalities, which it is easy to perceive must have an important influence on the medical evidence required in all future cases of infanticide, we are apt to lose sight of the principles upon which criminal jurisprudence, in relation to this crime, should be based. It cannot be disputed that *a living child* may be destroyed during birth, either before or after it has respired; or it may be destroyed after birth, but before what the law might deem an independent circulation has been established in its body. The wilful destruction of a child, under any of these circumstances, must be, morally speaking, as criminal as if it had been destroyed after it was entirely born, had fully breathed, and was completely separated from its mother. The English law, however, does not recognize the destruction of a child *during birth* as a case of infanticide, not even where the clearest evidence of life,—namely, the distension of the lungs with air from respiration, is afforded. In the case of R. *v.* Senior, it was decided that, if a mortal wound were inflicted on a child during birth, and before it had breathed, it would be murder, if the child were afterwards born alive, and died from the effects of the violence. But this decision does not affect the question which we have been examining. We contend that the legal completion of the crime should not be made contingent upon the child being *born alive;* for this is equal to making the crime and punishment of an offender to depend upon the accidental circumstance of her not having employed sufficient violence to effect her murderous purpose during birth. Besides, it is demanding evidence which must appear not only unnecessary in a moral point of view, but such as medical science can rarely be capable of affording.

In treating of the sinking of the lungs, as furnishing proof of a child being still-born, Dr. Cummin does not omit to point out the circumstances which may give rise to fallacy in the experiment. The means of diagnosis, in doubtful cases, are also very fairly described. We suspect that instances of the *healthy lungs* sinking, either "in their totality" or when divided into small pieces, are more common than our author is inclined to admit, in regard to children that have survived their birth. To form an opinion on this point, of course those cases only the history of which is known can be taken. We have lately met with an instance in which a child survived its birth many hours; but its lungs, which were healthy, had preserved all the characters of the fœtal state; they were livid, not crepitant, and had a sp. gr. of 1.046. When the organs were divided into thirty pieces, and placed in water, every piece sank equally to the bottom of the vessel. The sinking of the lungs, whether entire or divided, must not be looked upon as affording positive evidence of a child being born dead. In the case of R. *v.* Brain, Judge Park held that a child might be *born alive*, and not breathe for some time after its birth. This decision we conceive to be founded on good physiological principles, and the medical witness will perceive by it that, because the lungs of a child sink in water, he must not hastily abandon the investigation. Other circumstances may show that the child was actually alive when it sustained violence: the hydrostatic test is, indeed, at best only one of the sources of proof; and we see, by the occurrence of cases of this description, that it is one upon which the practitioner cannot always rely.

After a brief sketch of the peculiarities of the fœtus, and the changes which take place in the circulatory apparatus after birth, as furnishing corroborative evidence of extra-uterine life, Dr. Cummin enters upon the description of the causes of the death of the child. These are divided into natural and violent. It is highly important that a medical witness should be well acquainted with these; for, after having established that the child was born alive, it is equally necessary for him to show whether its death proceeded from violent or from natural causes. Further, as marks of violence may have an accidental origin, so, in order to support the charge of murder, must he be prepared to prove that the violence was criminally inflicted, and that it could not have originated from any accident to the child, either before, during, or after its birth. We must refer our readers to the work itself for an account of the means which the medical jurist now has at his command to establish these points; and a knowledge of which can alone enable him to triumph over the difficulties that legal ingenuity may endeavour to throw in his way.

We cannot take our leave of Dr. Cummin without congratulating him on the very able manner in which he has treated a subject of the first importance in medical jurisprudence.* Many questions relative to infanticide have been for a long time the opprobria of medical science; and we cannot deny that prejudice has had too wide an influence on the minds of some members of our profession to allow them to regard the sub-

* Since this was written, Dr. Cummin, we regret to say, has paid the great debt of nature. He died at the early age of thirty-seven.—Ed.

ject dispassionately. We consider that medical practitioners are deeply indebted to Dr. Cummin for having furnished them with the means of rebutting the specious and ill-founded views which have been so frequently and so successfully urged as objections to the employment of the hydrostatic test. These, we regret to find, are still, on too many occasions, eagerly laid hold of by barristers for the purpose of procuring the acquittal of their clients. We trust, however, that this judicious republication of Dr. Hunter's tract, with all the facts relative to the medico-legal history of infanticide which have accumulated during the last half century, will have the effect of undeceiving those who may still adhere to views which can neither be justified nor defended. We recommend this work to all who may be engaged in medico-legal investigations; and where is the practitioner who is certain of escaping from these? The easy and popular style in which the book is written renders it also well adapted to the barrister.

Dr. SCHWÖRER's pamphlet, it appears, is the substance of an inaugural lecture which the author delivered about two years previously, before the Academy of Freiburg. His chief object is to show that natural causes may sometimes produce marks of great violence on the body of the child during parturition. Having stated the points to which the medical jurist has to confine his attention in a case of suspected child-murder, he warns him against relying too confidently upon the dicta of those who deservedly hold a high reputation in the profession. As an instance of the fallacies into which a practitioner may thus be led, he quotes a statement of Haller's, in which this writer remarks that "fractures of the cranium in new-born children furnish evidence of external violence; and," observes our author, "not content with this affirmative declaration, he immediately afterwards asserts that such fractures never occur from natural causes." (P. 7.) Dr. Schwörer devotes but a slight space to the consideration of the proofs relative to the life of the child after birth: indeed, this does not appear to have been the object which he had in view. He enters, as it seems to us, rather more fully than he need have done into the psychological condition of women during delivery, and the possibility of the occurrence of a sudden attack of puerperal mania at this period, under which a female may become the unconscious murderess of her offspring. He brings forward numerous ingenious explanations of circumstances, which would, in general, be considered to furnish strong presumptive evidence against a mother on these occasions. His language bears the stamp of candour and philanthropy; for, while he loses no opportunity of endeavouring to reconcile the most adverse facts with the assumed innocence of a female, he does not withhold or conceal those points of evidence for which the medical witness should look, in order to establish the charge against her.

The design of the author being to prove that fractures of the fœtal cranium may occur *spontaneously during natural delivery*, a point which is certainly of great importance to be established, while it is of somewhat rare occurrence, we shall devote a short space to his remarks on the subject. Having first quoted the opinions of some old writers on the possibility of such accidents occurring during births, independently of external violence, he refers to actual cases, reported by Schmitt, Meissner, Jörg, and others; which we omit, in order to give a brief account of that which occurred to Dr. Schwörer himself.

A female, aged thirty-two, of delicate habit, was delivered on the 14th June, 1831, in the presence of the Professor and several of his pupils, of a male child. The labour was difficult, and lasted twenty-seven hours. This woman had gone eight days beyond the time at which, by calculation, she ought to have been delivered. During delivery, she became twice unconscious. The head of the child advanced slowly, in consequence of the resistance which it met with at the outlet. After it had descended, the pains suddenly ceased for an hour. Instruments were sent for; but, in the mean time, the child was suddenly expelled, and the Professor received it in his hands, so that it did not come in contact with any hard surface. The child did not breathe, but the pulsation of the heart could be felt, and there were present other satisfactory signs of life: it was found, however, impossible to resuscitate it. The body of the child was afterwards submitted to a close inspection. It was of the male sex, and perfectly mature. We shall confine our observations to the appearances presented by an inspection of the head. The skin over the right parietal bone was livid and swollen; and, on making an incision into it, a quantity of dark-coloured blood was found extravasated beneath. The cranium was examined more closely, and it was then found that the right parietal bone was fractured, or rather fissured, in two places. The first fissure was about an inch long, having a direction from the lambdoidal suture, about the middle of the posterior edge of the parietal bone, towards the centre of that bone. The second fissure was smaller; it commenced in the sagittal suture, near the upper and posterior angle of the parietal bone, passing downwards and outwards at nearly right angles to the suture. Blood was found extravasated between the dura mater and tunica arachnoides, in situations corresponding to these fissures.*

Dr. Schwörer seems to think that, had the history of this case remained unknown, and had a medico-legal opinion been required respecting the death of the child, the decision would most probably have been, that the child had survived its birth, notwithstanding the want of evidence of respiration; and further, that its death was owing to violent injury to the head. The unconsciousness under which the female laboured during delivery would have prevented her from giving any satisfactory account of the origin of the violence; and thus he leaves us to presume that she would have been condemned as the murderess.

We consider this case deserving of the attention of medical jurists of all countries. Facts of this description are always worthy of being recorded, as they serve to create caution in the expression of an opinion relative to the origin of marks of violence on new-born children. But we, for our parts, should not anticipate the evil consequences predicted by Dr. Schwörer on the occurrence of such a case. In this country, at least, the investigation, supposing nothing relative to the birth had been known, would not certainly have gone beyond a coroner's inquest. The child would have been pronounced still-born: we doubt whether any practitioner would, upon the evidence, have given an opinion that it had survived its birth. In our view, the death of this child was as much to be ascribed to the violence which the head sustained, as to the protraction of the labour. The origin of the violence is another question. It is here

* A striking example of a similar injury is recorded in our present Number, by Dr. Michaelis. (See Foreign Selections, sect. MIDWIFERY.)

proved to have taken place from spontaneous causes; but, at the same time, from the inspection it appears to have been of so slight a nature as scarcely to justify the grave fears entertained by the reporter. In general, medical witnesses make full allowance for the occurrence of accidental injuries to the child during delivery; and this certainly does not appear to us to have been a case in which this rule would have been departed from.

Dr. Schwörer's essay is well written; it carries on it the impress of a humane and reflecting mind. We could have wished that he had treated his subject in less figurative language, and that he had avoided quoting poetry.

Dr. JOHN BECK'S "*Researches*" consists of essays on various subjects; but nearly two-thirds of the volume are devoted to an examination of the questions connected with infanticide, under which head our author includes *criminal abortion*, as well as *child-murder*. We shall commence with a review of his opinions relative to abortion.

We must, in the outset, warn our readers that a very different interpretation is put on the meaning of the words Criminal Abortion in America and England. The laws of these countries differ materially from each other in the degree of medical proof which they require to establish the crime. Thus, in the State of New York, the higher offence is deemed complete only when the death of the child or the mother follows the attempt: hence Dr. Beck defines the crime,—the *murder of the fœtus in utero;* and the American medical jurist must be prepared to show that the life of the child or the mother was really destroyed by the means used. In England, the death of either is not required to be proved; if a fatal result follow, it can constitute no part of the evidence against a prisoner on a trial for abortion, although it may give rise to a subsequent charge of murder.

The author appears to us to give himself much unnecessary trouble to show that the opinions of the ancients respecting the animation of the fœtus are erroneous. Every modern physiologist now knows the exact value which is to be attached to these antiquated views. The *signs of abortion*, both in the living and dead subject, are very well described; but we think Dr. Beck has inadvertently fallen into an error respecting the nature of the medical evidence required on these occasions. Thus in speaking of uterine hydatids, as liable to give rise to ambiguity in a diagnosis, he says: "If hydatids are always the result of a degenerated conception, then the fact of impregnation is conceded; and this, after all, is the great point to be established in these cases." (P. 42.) Again, in speaking of moles, under the same circumstances, we find him asserting, that "If the mole be the product of a real conception, the great object of the investigation is at once conceded." (P. 43.)

Now, admitting that hydatids and moles are never met with in the uterus, except when there has been previous sexual intercourse, a point which yet remains to be proved, at least with respect to hydatids, we cannot assent to Dr. Beck's sweeping statement, that "the great object of the investigation is at once conceded." In a case of criminal abortion, the medical evidence must be directed to the proof of actual pregnancy, or of delivery, if it have followed: evidence of previous intercourse is neither admitted nor required. The real question is, whether a woman was with child or not at the time of the attempt; not whether she had previously exposed herself to the chance of impregnation. Let us imagine then, that, in a particular case, a mole or hydatids had been expelled from the uterus, and that the woman was not really pregnant, the charge of criminal abortion could not be sustained; since the most material proof, namely, the fact of her being *with child*, would be wanting.

The section on the criminal means for "destroying the fœtus" (procuring abortion,) appears to us unnecessarily long. In speaking of the secale cornutum, the author mentions a case which occurred to him where a woman took three drachms of this substance for the purpose of producing abortion, but without any effect. Dr. Beck does not seem to be aware of the difference in the strength of this drug which Kluge of Berlin has shown to exist in it, according as it is collected before or after harvest.

A very considerable space is devoted to the subject of infanticide, or child-murder; but we cannot compliment the author on the manner in which he has treated it. He has allowed himself to be swayed too much by the opinions of others, and he seems throughout to have trusted too little to his own means of observation. In consequence of this, we meet with several errors which may have the effect of bewildering or of misleading the medical witness; and we, therefore, conceive it to be our duty to point them out, in order that they may be corrected in a future edition of the work. In commencing his subject, we find him making this extraordinary statement:

"In a medico-legal point of view, no child ought to be considered as capable of sustaining an independent existence until the seventh month (of gestation) has been fully completed. Accordingly, if it can be proved that the child which is the subject of investigation has not attained this age, no charge of infanticide can or ought to be entertained." (P. 68.)

We have known an ignorant coroner in this country refuse to hold an inquest on the body of a child under the seventh month, because it was laid down by some law-authority that children *could not be born alive* under that age. Now, we have to observe that the law of England, and we presume, from Dr. Beck's subsequent remarks on American legislation, the same holds in the States, does not require that a child should be capable of sustaining an independent existence in order to render its wilful destruction after birth murder. Indeed, it is absurd to suppose that the destruction of a child born alive under the seventh month of pregnancy would be suffered to pass unpunished in either country. We have, in our own experience, known a child, born between the sixth and seventh month, live a fortnight; and certainly cases are not unfrequent in which children brought into the world at this early period survive some hours. But, because these immature beings are not likely to maintain an independent existence, is the killing of them to be permitted to take place with impunity, in a country which pretends to any degree of civilization? If not, how can Dr. Beck justify the position that a charge of infanticide neither *can* nor *ought* to be entertained when the child has not reached the seventh month?

Some remarks are made respecting the chemical composition of the fœtal blood, as furnishing evidence of live birth or the contrary. These might have been well omitted: the differences in the blood of the child before and after respiration are slight: they require time for their development, and the most careful chemical analysis for their detection. In the generality of cases of infanticide, the child being destroyed in the act of birth, or immediately afterwards, there can be no appreciable difference in the chemical composition of this liquid. There follows a very good account of Bernt's observations on the changes in the foramen ovale and ductus arteriosus after birth; but we are inclined to think that the author has had no practical experience on this subject, or he would not have hazarded the assertion:—

"If, therefore, *the ductus arteriosus be found cylindrical in its shape, and not contracted towards the aorta, and if it equal in size the trunk of the pulmonary artery,* the inference would be that the child was not born alive." (P. 76.)

The italics are the author's own: we shall only observe, that, if this statement were relied on, it would give rise to the gravest errors in practice. We have lately met with two cases in which the ductus arteriosus had all the characters here represented, and yet both of these children were born alive: one survived its birth half an hour, and the other twenty-four hours. Besides, the charge is not contingent upon a child being *born* alive, but upon the degree of perfection with which the respiratory process has been performed. There are several other errors in this part of the essay which we shall pass over, because they are unimportant and can have but little effect on medical evidence. One statement, however, must be alluded to, as a reliance on it would positively place the life of an accused female in danger. It is in relation to that frequent occurrence,—"the presence of ecchymosis or extravasation of blood on the body of the child." "This," observes Dr. Beck, "is the last sign to show that the blood has circulated after birth." (P. 94.) We are at a loss to conceive how such an opinion could have been expressed, since it requires but a very limited experience to know that extravasations and ecchymoses of large extent may not only be formed on the body of the child before birth, but before respiration is established. How, therefore, can these marks show that the blood has circulated *after birth*? They furnish good evidence of the child having been alive when they were received; but they afford no proof of its having been born alive.

The author discusses largely the value of the hydrostatic test; but he falls into the error of asserting "that, when the lungs of a child float in water, it must have respired, and *therefore must have been born alive.*" (P. 101.) We have already entered fully into this question, and we shall therefore decline saying more on the subject. Dr. Beck will excuse us for telling him that, "when lungs which have actually respired are cut into," the incisions are not always followed by a flow of blood. In order that this should be observed, respiration must be much more completely established than the examiner is likely to find it in most cases of child-murder. So, again, instead of quoting Bernt, as to the effect of air artificially introduced into the lungs, (99,) in producing a change of colour in those organs, we would advise him to perform the experiment himself, when he will find that, according to the manner in which the inflation is accomplished, their colour will resemble more or less closely that produced by natural respiration. We must also protest against the assertion, that when "a child has been born dead, the arteries and veins of the lungs are found *destitute of blood,* and in a collapsed state," (108;)

for it is by no means unusual to find blood in the pulmonary vessels of children which have been born dead without respiring.

We should have scarcely supposed that Dr. Beck would have found it worth his while to argue the question as to whether a child can breathe during birth, while its head is protruding; or to quote authorities in support of a fact with which we presume most professional men must be familiar. We agree with him that the death of a child, under these circumstances from natural causes, is not very probable; and we approve of the judicious summary which he has made of these causes. Still, the medical jurist must remember that a child may perish on such occasions, independently of all criminal violence.

In taking our leave of Dr. Beck, we assure him it is with regret that we have felt ourselves obliged to point out what we consider to be serious errors in his volume. We find ourselves the more imperiously called on to do so, because the Essay on Infanticide, although now separately published, forms a part of his brother's admirable "Elements of Medical Jurisprudence;" a work of most deserved authority, and very extensively circulated among the members of the profession both in this country and America. The great defects, however, of the Essay on Infanticide, are its want of arrangement and its redundancies. By these the author has in a considerable degree destroyed its utility as a practical guide to the medical witness; for it is by this rule, after all, that our author must submit to be tried. That Dr. Beck is a man of most extensive erudition will at once appear from a perusal of his work. He quotes authorities without number from all countries and on all subjects, whether directly or indirectly bearing upon the matter which he is discussing. It is thus, in an earnest endeavour to prevent his work from appearing deficient in facts or reasonings, that he has needlessly burthened it with details which, we are sure, slight reflection would prove to him to be unnecessary.

---

